# Roles of Fatty Acid Oversupply and Impaired Oxidation in Lipid Accumulation in Tissues of Obese Rats

**DOI:** 10.1155/2013/420754

**Published:** 2013-05-13

**Authors:** Nicholas D. Oakes, Ann Kjellstedt, Pia Thalén, Bengt Ljung, Nigel Turner

**Affiliations:** ^1^AstraZeneca R&D Mölndal, 431 83 Mölndal, Sweden; ^2^Diabetes and Obesity Program, Garvan Institute of Medical Research, Darlinghurst, NSW 2010, Australia; ^3^School of Medical Sciences, University of New South Wales, Sydney, NSW 2052, Australia

## Abstract

To test the roles of lipid oversupply versus oxidation in causing tissue lipid accumulation associated with insulin resistance/obesity, we studied *in vivo* fatty acid (FA) metabolism in obese (Obese) and lean (Lean) Zucker rats. Indices of local FA utilization and storage were calculated using the partially metabolizable [9,10-^3^H]-(R)-2-bromopalmitate (^3^H-R-BrP) and [U-^14^C]-palmitate (^14^C-P) FA tracers, respectively. Whole-body FA appearance (*R*_*a*_) was estimated from plasma ^14^C-P kinetics. Whole-body FA oxidation rate (*R*_ox_) was assessed using ^3^H_2_O production from ^3^H-palmitate infusion, and tissue FA oxidative capacity was evaluated *ex vivo*. In the basal fasting state Obese had markedly elevated FA levels and *R*_*a*_, associated with elevated FA utilization and storage in most tissues. Estimated rates of muscle FA oxidation were not lower in obese rats and were similarly enhanced by contraction in both lean and obese groups. At comparable levels of FA availability, achieved by nicotinic acid, *R*_ox_ was lower in Obese than Lean. In Obese rats, FA oxidative capacity was 35% higher than that in Lean in skeletal muscle, 67% lower in brown fat and comparable in other organs. In conclusion, lipid accumulation in non-adipose tissues of obese Zucker rats appears to result largely from systemic FA oversupply.

## 1. Introduction

Disturbances in fatty acid metabolism may cause several key features of the insulin resistance syndrome, including impaired glucose regulation, dyslipidemia, and obesity. Thus systemic free fatty acid (FA) oversupply can decrease insulin-stimulated glucose uptake in skeletal muscle [1, 2], reduce insulin suppression of hepatic glucose production [3], and alter glucose-stimulated insulin secretion [4]. Furthermore, an oversupply of FA to liver may cause dyslipidemia, including hypertriglyceridemia and the atherogenic lipoprotein profile [5]. With regard to FA utilization, reductions in mitochondrial content and diminished fatty acid oxidation capacity in skeletal muscle and adipose tissue have been linked with obesity and insulin resistance [6–14]. Additionally, in conditions of impaired insulin action there is also a reduced ability to appropriately switch between glucose and lipid fuels (i.e., metabolic inflexibility), postulated to play an important role in the development of obesity [15]. 

The obese Zucker rat is an animal model possessing major metabolic features seen in conditions of human insulin resistance, including glucose metabolic insulin resistance, hypertriglyceridemia, and elevated nonadipose tissue lipid levels [16, 17], which have been implicated in both the development of insulin resistance and lipoapoptosis in tissues including the pancreas and heart [18]. Inappropriate deposition of triglycerides and other bioactive fatty acid metabolites in a tissue may result from a systemic oversupply of fatty acid or from a local defect in fatty acid oxidation. The obese Zucker rat has a loss-of-function mutation in the leptin receptor, and although leptin has been shown to enhance local rates of lipid oxidation [19, 20], the relative contribution of reduced FA utilization to the lipotoxic state observed in tissues of these animals *in vivo* is still not well established. 

We were interested in elucidating the mechanisms of lipid accumulation in non-adipose tissues of obese Zucker rats: oversupply or underutilization? The aim of this study was therefore to determine how the fluxes and metabolic fate of FA are altered in obese versus lean Zucker rats. This was done in three independent experimental series. Series 1 quantified FA uptake and metabolic fate *in vivo* at the individual tissue level based on the simultaneous combined use of [9,10-^3^H]-(R)-2-bromopalmitate (^3^H-R-BrP) and [U-^14^C]-palmitate (^14^C-P). The ^3^H-R-BrP tracer is used to estimate local FA (oxidative + nonoxidative) utilization while ^14^C-P is used to assess non-oxidative FA disposal into lipid stores [21]. Series 2 examined FA oxidation and its dependence on FA levels, using nicotinic acid as an antilipolytic agent. Finally, Series 3 was used to assess FA oxidation capacity in a comprehensive range of metabolically important tissues *ex vivo*. Our results give strong support for the hypothesis that the major factor responsible for tissue lipid accumulation in obese Zucker rats is increased plasma FA availability. 

## 2. Materials and Methods

### 2.1. Animals

Experimental procedures were approved by the local ethics review committee on animal experiments (Göteborg region). Male 8-week old Lean (FA/FA) and obese (fa/fa) Zucker rats (Charles River Wiga GmbH, Suffield, FRG) were maintained in a temperature controlled (20–22°C) room with a 12 h light-dark cycle (lights on at 06:00) and free access to rodent chow (R3 Laktamin AB, Stockholm, Sweden) and tap water.

### 2.2. Acute Study Preparation for Series 1 and 2

At 07:00 on the morning of the study food was withdrawn. Then at 09:00 the rats were anaesthetized with Na-thiobutabarbital (Inactin, RBI, Natick, MA), with the lean and obese rats receiving 120 and 180 mg kg^−1^ (I.P.), respectively. Body temperature was monitored using a rectal probe and maintained at 37.5°C throughout the experiment. Animals were tracheotomized and catheters were placed in the right jugular vein for tracer administration and left carotid artery for continuous monitoring of arterial blood pressure and heart rate and for blood sampling via a device allowing minimum sample volumes. Arterial catheter patency was maintained by continuous infusion (10 *μ*L min^−1^) of a sterile saline solution containing sodium citrate (20.6 mmol L^−1^). 

### 2.3. Series 1: Tissue-Specific FA Metabolism *In Vivo*

#### 2.3.1. Unilateral Hindlimb Muscle Contraction

Immediately following catheterization (described above), the sciatic nerves were exposed and cut bilaterally at the gluteal level. Unilateral electrical sciatic nerve stimulation was applied with ring electrodes at 0.5 Hz to induce sustainable twitch contractions in muscles of one hind leg as described previously [22].

#### 2.3.2. Tracer Preparation

^3^H-R-BrP tracer was synthesized and purified using methods described in [23]. Tracer infusates were prepared freshly each day. For each rat ~5 × 10^7^ dpm ^3^H-R-BrP and ~2.5 × 10^7^ dpm ^14^C-P (Amersham, Solna, Sweden), as well as 152 nmol Na-palmitate (Sigma, St. Louis, MO), were complexed to essentially fatty acid-free bovine serum albumin (BSA) (Sigma) as detailed in [22]. *Protocol.* Unilateral sciatic nerve stimulation was commenced 70 min after completion of surgical preparation and 20 min prior to commencing tracer administration. All blood samples were collected via the carotid catheter into K-EDTA containing tubes (Microvette CB300, Sarstedt, Nümbrecht, Germany) via a device designed to reduce sample volume. Immediately before tracer infusion a 200 *μ*L basal blood sample was collected for determination of plasma insulin and substrate levels. Tracer administration and blood sampling were performed according to previously described methods [22]. Briefly, the albumin-palmitate-tracer complex was infused through the jugular catheter at 230 *μ*L min^−1^ for 4 min. Blood samples were centrifuged immediately at 4°C and a 25 *μ*L plasma aliquot placed directly into lipid extraction mixture (described in [22]) for determination of plasma ^3^H-R-BrP and ^14^C-P concentrations. After collection of the final blood sample, 16 min after commencing the tracer infusion, rats were killed with an overdose of thiobutabarbitol (120 mg kg^−1^). Tissues were collected and samples (~100 mg) were combusted for determination of total ^3^H and ^14^C content [22]. *Calculations.* The clearance rate of ^3^H-R-BrP by an individual tissue (*K*_f_*), an index of the ability of the tissue to utilize FAs, was calculated as previously described [22], as

(1) $$\begin{array}{c} K_{f}^{*}=\frac{\;m_{B}}{\int_{0}^{T}c_{B}\left(t\right)dt}, \end{array}$$

where *T* is the time of tissue collection (16 min), *m*_*B*_ is the total tissue ^3^H content (at *t* = *T*), and *c*_*B*_ is the arterial plasma concentration of ^3^H-R-BrP. An index of FA utilization rate (*R*_f_*) was calculated as

(2) $$\begin{array}{c} R_{f}^{*}=C_{P}\times K_{f}^{*}, \end{array}$$

where *C*_*P*_ is the arterial plasma FA concentration. 

An index of the clearance of ^14^C-P into storage products (*K*_*fs*_) was calculated as

(3) $$\begin{array}{c} K_{fs}^{}=\frac{\;m_{P}}{\int_{0}^{T}c_{P}\left(t\right)dt}, \end{array}$$

where *m*_*P*_ is the total tissue ^14^C content (at *t* = *T*) and *c*_*P*_ is the arterial plasma ^14^C-P concentration. This assumes that all of the ^14^C label originating from locally activated ^14^C-P directed into oxidative metabolism would be lost from the tissue (largely as ^14^CO_2_) by the time of tissue sampling. An index of the rate of FA incorporation into storage (*R*_*fs*_) was calculated as

(4) $$\begin{array}{c} R_{fs}=C_{P}\times K_{fs}. \end{array}$$

Assuming certain conditions are met [22], *R*_f_* is proportional to the genuine rate of FA utilization (*R*_*f*_), that is,

(5) $$\begin{array}{c} R_{f}^{*}={\text{LC}}^{*}\times R_{f} \end{array}$$

with a constant of proportionality (“lumped constant”) LC*. Since *R*_*f*_ is the sum of oxidative disposal (*R*_*f*_ox__) and non-oxidative disposal (*R*_*fs*_),

(6) $$\begin{array}{c} R_{f_{\text{ox}}}={{\text{LC}}^{*}}^{-1}\times R_{f}^{*}-R_{fs}. \end{array}$$

Note that when *R*_*f*_ox__ = 0, LC*^−1^ = *R*_*fs*_/*R*_f_*. We have previously suppressed fatty acid oxidation using pharmacological *β*-oxidation inhibition to obtain crude estimates of LC* for different tissues in the rat [22]. LC* and the reliability of the *R*_*f*_ox__ values derived vary in a tissue-specific manner [22] with the following values assumed for the present work: skeletal muscle 0.27 and heart 0.19.

Estimates of whole-body plasma ^14^C-P clearance (*K*_*P*_) were calculated according to [22]. 

#### 2.3.3. Determination of Plasma ^3^H-R-BrP and ^14^C-P Concentrations, as well as Tissue ^3^H and ^14^C-Levels

Plasma ^3^H-R-BrP and ^14^C-P were resolved using an acid lipid extraction procedure. Total tissue ^3^H and ^14^C levels were determined by combusting tissue samples using a Packard System 387 Automated Sample Preparation Unit (Packard Instruments Co., Inc., Meriden, CT). These methods are described in detail in [22]. 

### 2.4. Series 2: Whole-Body FA Oxidation Rate and Its Dependence on Plasma FA Level

#### 2.4.1. Groups

Four groups (*n* = 2-3 per group) of both lean and obese Zucker rats were studied in order to generate a range of plasma FA levels: a vehicle control group receiving a normal saline infusion and three groups receiving intravenous nicotinic acid infusion at the doses of 10, 100 and 1000 nmol kg^−1^ min^−1^, respectively.

#### 2.4.2. Tracer Preparation

Tracer infusates, ~2 · 10^8^ dpm per rat [9,10 ^3^H] palmitic acid (^3^H-P, Amersham, Solna, Sweden) and 305 nmol Na-palmitate (Sigma, St. Louis, MO), were freshly prepared daily. The tracer and Na-palmitate were prepared in 150 *μ*L ethanol and added dropwise to 0.6 mL of continuously stirred 4% (w/v) essentially fatty acid-free bovine serum albumin (BSA, Sigma, St. Louis, MO) in normal saline. The infusate was made up to a final volume of ~3 mL per rat by addition of normal saline.

#### 2.4.3. Protocol

After a 2 h postsurgery recovery period, two basal blood samples (~150 *μ*L) were collected 15 min apart for analysis of plasma FA, TG, glucose, and insulin. Immediately following collection of the second blood sample, intravenous infusions of nicotinic acid (or vehicle) and tracer were started. The albumin-palmitate-^3^H-P complex was infused at a constant rate (~1 × 10^6^ dpm min^−1^, 17 *μ*L min^−1^). Arterial blood samples (~75 *μ*L) were collected 10, 20, 40, 60, 80, 100, and 120 min after the start of tracer infusion. For each sample, plasma was separated as quickly as possible in a refrigerated centrifuge. One 25 *μ*L aliquot was placed into 2 mL lipid extraction mixture, for determination of ^3^H-P and ^3^H_2_O; the remainder was used for analysis of FA level. After collection of the final blood sample, rats were killed with an overdose of thiobutabarbitol (120 mg kg^−1^).

#### 2.4.4. Measurement of Plasma Levels of ^3^H-P and ^3^H_2_O

To discriminate ^3^H-P from total plasma ^3^H activity, a lipid extraction and separation procedure was performed on plasma samples. This involved an initial acid lipid extraction using a mixture of isopropanol-heptane-1 mol/L acetic acid (40 : 10 : 1 vol) followed by solid phase separation of free fatty acids (including ^3^H-P) from neutral lipids. ^3^H_2_O was estimated as the ^3^H-activity in the lower (isopropanol-water) phase of the lipid extraction procedure.

#### 2.4.5. Rates of Plasma FA Appearance (*R*_*a*_) and Oxidation (*R*_ox_)

Plasma FA mobilization was assessed using a constant infusion of ^3^H-palmitate (^3^H-P). After attainment of isotopic steady states (<40 min after the start of tracer infusion), the plasma clearance rate of ^3^H-P (*K*) was calculated as

(7) $$\begin{array}{c} K_{}=\frac{i_{p}}{c_{p}\left(\infty \right)}, \end{array}$$

where *i*_*p*_ is the tracer infusion rate (dpm min^−1^) and *c*_*p*_(*∞*) is the steady state arterial concentration of ^3^H-P (dpm mL^−1^). The rate of appearance of plasma FAs (*R*_*a*_) was calculated as

(8) $$\begin{array}{c} R_{a}=C_{p}\times K, \end{array}$$

where *C*_*p*_ is the arterial plasma FA concentration (*μ*mol mL^−1^).

Appearance of ^3^H_2_O in the plasma was linear from the earliest time point throughout the study consistent with rapid attainment of steady state of the labeled oxidation precursor pool. The fraction of plasma FA undergoing oxidation (*f*_ox_) was estimated using the relationship

(9) $$\begin{array}{c} f_{\text{ox}}=\frac{V_{w}\times dc_{w}/dt}{i_{p}}, \end{array}$$

where *V*_*w*_ is the total water space of the rat (estimated from body weight, BW in g, using separate regression equations: for obese animals % water = 59.3 − 0.027 × BW and for lean animals % water = 73.4 − 0.030 × BW obtained from in-house water content analyses of obese and lean Zucker rats, resp.), *c*_*w*_ is the plasma concentration of  ^3^H_2_O, and *t* is the time from commencement of tracer infusion. The derivative above was estimated from the slope obtained from linear regression analysis of the ^3^H_2_O plasma versus time data for the period *t* = 10 to *t* = 120 min. An estimate of the whole-body rate of FA clearance into oxidation (*K*_ox_) was calculated as

(10) $$\begin{array}{c} K_{\text{ox}}=f_{\text{ox}}\times K. \end{array}$$

The rate of whole-body FA oxidation (*R*_ox_) was calculated as

(11) $$\begin{array}{c} R_{\text{ox}}=f_{\text{ox}}\times R_{a}. \end{array}$$

### 2.5. Series 3: *Ex Vivo* Fatty Acid Oxidation

Fatty acid oxidation was measured in tissue homogenates using a modified version of a previously published method [24]. Briefly, tissues were homogenized in either 9 volumes (epididymal white adipose tissue; WAT), 19 volumes (cerebellum), or 39 volumes (heart, brown adipose tissue (BAT), liver, red gastrocnemius, and white quadriceps) of ice-cold 250 mmol L^−1^ sucrose, 10 mmol L^−1^ Tris-HCl, 1 mmol L^−1^ EDTA. For assessment of palmitate oxidation, 50 *μ*L of tissue homogenate was then incubated with 450 *μ*L reaction mixture (pH 7.4). Final concentrations of the reaction mixture were (in mmol L^−1^) 100 sucrose, 10 Tris-HCl, 5 potassium phosphate, 80 potassium chloride, 1 magnesium chloride, 2 malate, 2 ATP, 1 dithiothreitol, 0.2 EDTA, 2 L-carnitine, 0.05 coenzyme A (CoA), 0.2 palmitate [+0.5 *μ*Ci 1-^14^C-palmitate], and 0.3% (w/v) fatty acid-free BSA. After 90 min of incubation at 25°C, the reaction was stopped by the addition of 100 *μ*L of ice-cold 1 mol L^−1^ perchloric acid. CO_2_ produced during the 90 min incubation was collected in 100 *μ*L of 1 mol L^−1^ sodium hydroxide. ^14^C counts present in the acid-soluble fraction were also measured and combined with the CO_2_ values to give the total palmitate oxidation rate.

### 2.6. Plasma Insulin and Substrate Concentrations

Insulin concentrations were determined using radioimmunoassay (rat insulin RIA kit; Linco Research, St. Charles, MO). Colorimetric kit methods were used for the measurement of plasma FA (NEFA C; Wako, Richmond, VA), triglycerides (Triglycerides/GB; Boehringer Mannheim, Indianapolis, IN), and glucose (Glucose HK; Roche, Stockholm).

### 2.7. Statistics

Differences between Lean and Obese groups were assessed using Student's *t*-tests, assuming equal group variance. Systematic between-group differences in muscle parameters were assessed using 2-way analysis of variance (ANOVA) using the program SPSS (SPSS, Chicago, IL). Linear regression analysis was performed using GraphPad Prism (GraphPad Software Inc., La Jolla, CA). Results are reported as mean ± SE. *P* < 0.05 was considered statistically significant.

## 3. Results

### 3.1. Series 1: Tissue-Specific FA Metabolism *In Vivo*

Body weights and general plasma factors for Series 1 animals are summarized in Table 1. Estimates of body composition, lean and fat mass, have also been made as previously described [25]. As expected, obese Zucker rats weighed approximately 50% more than age-matched lean Zucker rats, due to increased fat mass, and displayed hyperinsulinemia, hypertriglyceridemia, and a mild hyperglycemia.

**Figure 1:**
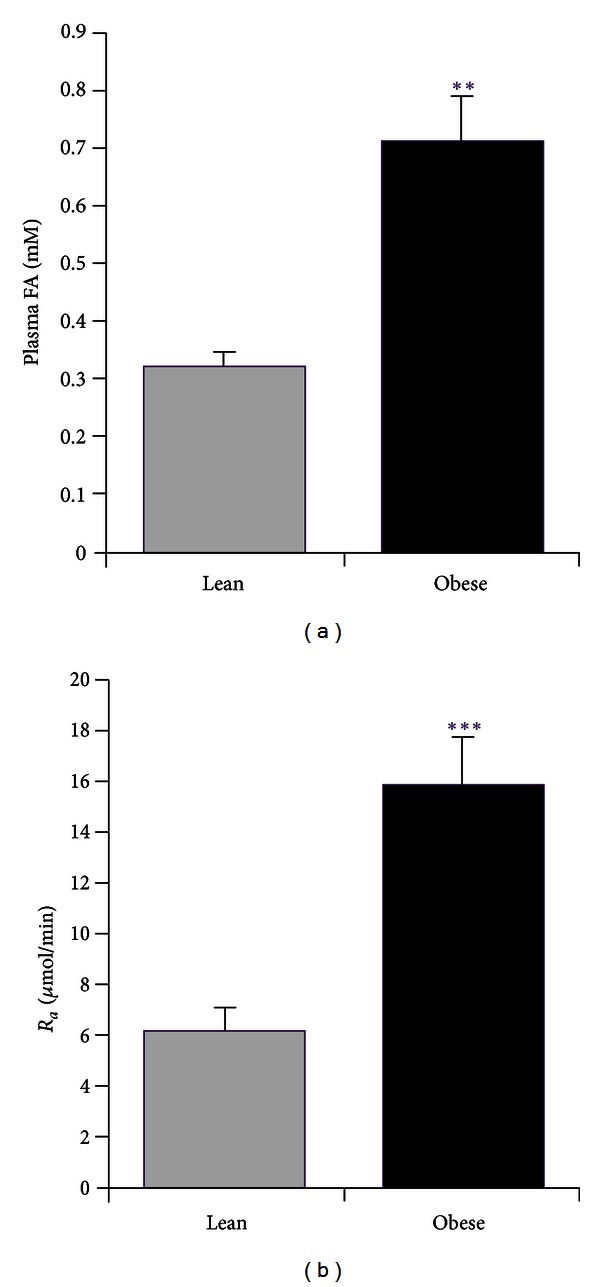
Whole-body FA metabolism in lean (Lean) and obese (Obese) Zucker rats. *R*_*a*_ plasma FA appearance rate. Results are expressed as mean ± SE (*n* = 6 rats per group). **P < 0.01, ***P < 0.001 versus Lean.

Plasma FA level and rate of appearance of FA (*R*_*a*_), calculated from the ^14^C-palmitate kinetics, are presented in Figure 1. Obese animals had substantially elevated systemic FA availability, compared to lean Zuckers, due to an elevated rate of entry of FA into the plasma as shown by the *R*_*a*_ data. Metabolic clearance rates of ^14^C-palmitate (*K*_*P*_, expressed per rat) were similar in lean and obese animals, despite the much greater total tissue mass of the obese animals (data not shown).

The rate of ^3^H-R-BrP clearance from plasma into a tissue (*K*_f_*) provides an index of the local ability to utilize FA for both oxidative and non-oxidative metabolism (storage), independent of the direct influence of plasma FA level. Tissue-specific clearance of ^14^C-palmitate into storage (*K*_*fs*_) indexes the local ability to store plasma FA. Independent of group, *K*_f_* and *K*_*fs*_ had a range of 2 orders of magnitude across the different tissues sampled, with cerebellum having the lowest and liver the highest values (Table 2). Also independent of group is the large difference between adipose tissue types with BAT having a much greater ability to take up and store FA than WAT. Comparing results for obese and lean animals, there were no differences in *K*_f_* or *K*_*fs*_ values for liver, cerebellum, or WAT. In BAT and heart *K*_f_* was lower in the obese compared to the lean animals, while *K*_*fs*_ was similar in the two groups. This indicates a reduced ability to metabolically sequester available FA and a preferential diversion towards non-oxidative disposal in these tissues of obese compared to lean Zuckers. 

Hindlimb muscle *K*_f_* and *K*_*fs*_ results are summarized in Table 3. Results for five corresponding muscles from both hind legs are presented: one leg subject to repetitive efferent electrical stimulation of the sciatic nerve (Stim-Leg) versus the unstimulated control leg (Con-Leg). Examining first the results in the quiescent control leg muscles, it is apparent that independent of group, *K*_f_* and *K*_*fs*_ roughly rank according to expected oxidative capacity with glycolytic muscle (WQ and WG) < intermediate mixed fiber type muscle (EDL) < highly oxidative muscle (RG and RQ). There was no systematic difference in *K*_f_* in the quiescent muscles between lean and obese groups. *K*_*fs*_ did however tend to be modestly higher (by 15%, *P* < 0.01, ANOVA) in the quiescent muscles of the obese compared to the lean animals. These results suggest a similar ability to metabolically sequester available FA in the two groups but that in the obese animals there was a slight preference for disposal of FA into non-oxidative metabolism compared to the lean animals. 

Electrical stimulation of the sciatic nerve at the gluteal level induced twitch contractions in the lower leg muscles (WG, EDL, and RG) but not in the thigh muscles (WQ and RQ). Correspondingly, for each lower leg muscle the *K*_f_* value was significantly higher in the Stim-Leg versus Con-Leg, while there were no differences in *K*_f_* between the Stim-Leg and Con-Leg for the 2 noncontracting thigh muscles (Table 3). The average contraction-induced increase in lower leg muscle *K*_f_* was similar in both lean and obese groups (group effect, *P* > 0.05, ANOVA). Sciatic nerve stimulation tended to induce a small increase in *K*_*fs*_ (3 out of 3 muscles in lean and 1 out of 3 muscles in obese, Table 3) which when averaged over all muscles was not significantly different in lean versus obese groups (*P* > 0.05, ANOVA). Altogether, the relatively much larger contraction induced increase in *K*_f_* than *K*_*fs*_ (in both groups) indicates that the contraction-induced increase in FA clearance is almost exclusively diverted into oxidation.

Parameters reflecting *in vivo* metabolic fluxes of plasma FA in individual tissues are given in Tables 4 and 5. *R*_f_* indexes the total rate of plasma FA utilization into both oxidative and non-oxidative metabolism. *R*_*fs*_ is an estimate of the rate of plasma FA incorporation into storage (non-oxidative metabolism) only. Obese animals had substantially higher *R*_f_* and *R*_*fs*_ values compared with Lean in the majority of tissues including cerebellum, liver, and WAT (Table 4), as well as all skeletal muscles examined (Table 5). This was caused by the higher FA levels in the obese compared to the lean animals resulting from the higher rate of entry of FA into plasma described above. Only in heart and BAT, was *R*_f_* similar in both Lean and Obese groups (Table 4). 

Contraction increased *R*_f_* and *R*_*fs*_, in the lower leg muscles, an expected consequence of the increases in *K*_f_* and *K*_*fs*_, respectively (referred to above). The extent of the increase in *R*_f_* was greater in the Obese compared to Lean group: (*P* < 0.05, group effect, ANOVA). 

**Figure 2:**
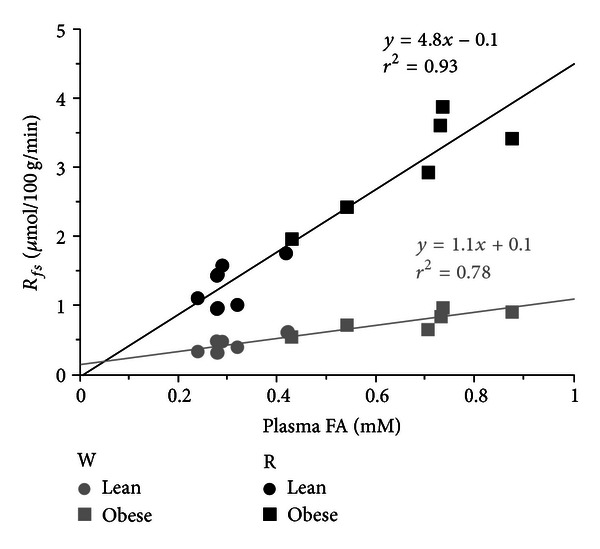
Relationship between *in vivo* flux of FA into storage in skeletal muscle and plasma FA level. Circles represent results from Lean, while squares represent results from Obese animals. Results for white quadriceps muscle (gray symbols, W) and red quadriceps muscle (black symbols, R) are plotted for individual animals (*n* = 6 rats per group). Straight lines represent linear regression equations.

Figure 2 shows the relationship between *R*_*fs*_ for WQ and RQ (Con-Leg only) and plasma FA level in individual animals. First it is apparent that in these quiescent muscles there is a simple linear dependence of plasma FA flux into storage on plasma FA concentration and that the same relationship seems to hold for both obese and lean groups, suggesting that the increased flux of FA into non-oxidative disposal in resting muscle in obese Zuckers is a direct result of the higher plasma FA levels. Second, the red oxidative muscle (RQ) has a much greater ability to store available FA than the glycolytic muscle (WQ) as evidenced by the greater slope in RQ compared to WQ (*P* < 0.0001). Linear relationships also apply for the other muscles (data not shown).

Tissue specific rates of FA oxidation cannot be calculated directly as the difference between *R*_f_* and *R*_*fs*_ because of the slower kinetics of  ^3^H-R-BrP compared with native FA. Plasma FA oxidation (*R*_*f*_ox__) can however be estimated indirectly from *R*_*fs*_ and *R*_f_* in some tissues including muscles (see Table 6) as described in the Materials and Methods section. *R*_*f*_ox__ in quiescent hindlimb muscles were low in comparison to the flux of FA into non-oxidative disposal (*R*_*fs*_) and generally similar in obese compared to lean Zuckers. The exception was in the RQ muscle where the levels were higher in Obese versus Lean. Contraction (only occurring in WG, EDL and RG) induced substantial increases in *R*_*f*_ox__ to levels similar in magnitude to the levels of *R*_*fs*_ in the corresponding muscles. Averaged across the three contracting muscles, the contraction induced increase in FA oxidation was similar in obese versus lean rats, 2.0 ± 0.2 versus 1.6 ± 0.3 *μ*mol/100 g/min (*P* > 0.05) respectively. Obese rats apparently also had similar rates of plasma FA oxidation compared to lean rats in two other contracting muscle tissues, diaphragm and heart. Thus diaphragm *R*_*f*_ox__ for Lean was 4.3 ± 0.7 versus Obese 5.9 ± 1.8 *μ*mol 100 g^−1^ min^−1^ while *R*_*f*_ox__ for the heart was for Lean 43.4 ± 6.1 versus Obese 42.2 ± 4.5 *μ*mol 100 g^−1^ min^−1^.

### 3.2. Series 2: Whole-Body FA Oxidation Rate and Its Dependence on Plasma FA Level

**Figure 3:**
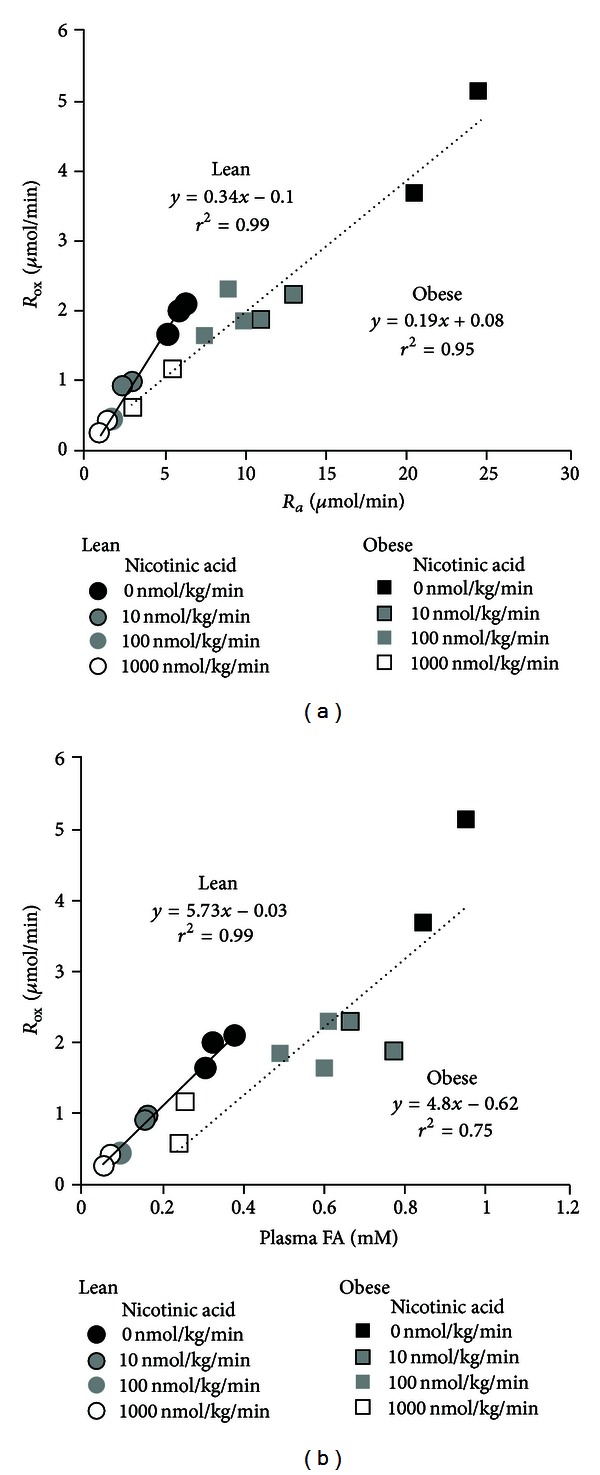
Dependence of FA oxidation (*R*_ox_) on both the rate of FA appearance, *R*_*a*_ (a), and FA level (b), in lean and obese Zucker rats. FA availability has been pharmacologically manipulated using the antilipolytic agent nicotinic acid to generate a range of FA levels (*n* = 2-3 rats per dose).

In Series 1, obese Zucker rats were observed to have a general elevation in plasma FA level and utilization at the whole body, as well as in muscle, fat, and liver compared to lean controls. To examine the dependence of FA oxidation on plasma FA availability, the antilipolytic agent nicotinic acid was used in several doses to suppress *R*_*a*_ in order to generate a range of FA levels that overlapped in the lean and obese animals.  Figure 3 shows the relationships between *R*_*a*_ and rate of FA oxidation (*R*_ox_) and plasma FA level, respectively. The nicotinic acid infusions applied succeeded in dose dependently reducing FA availability in both lean and obese Zucker rats. In both groups there was a tight linear dependence of *R*_ox_ on *R*_*a*_ (Figure 3(a)), with regression line intercepts virtually coinciding with the origin. The slope of this relationship in the obese Zucker was however 44% less than that in the lean animals (*P* < 0.01), indicating that under conditions of an equal rate of systemic FA supply the obese Zuckers would exhibit a reduced rate of FA oxidation compared to the lean Zuckers. Strong linear relationships were also apparent between *R*_ox_ and plasma FA level in both groups of Zucker rats (Figure 3(b)) with the regression line for the obese Zuckers having a lower intercept (*P* < 0.05) and tending to have a lower slope than the line for the lean Zuckers. Thus, under conditions of comparable FA levels, the obese Zuckers would manifest a reduced FA oxidation rate compared to lean Zuckers. 

### 3.3. Series 3: *Ex Vivo* Fatty Acid Oxidation

**Figure 4:**
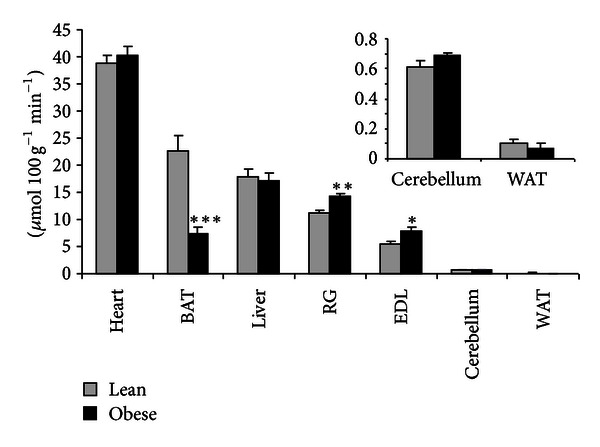
FA oxidation capacity of individual tissues, determined *ex vivo*, based on the ability of tissue homogenates to oxidize palmitate. Results are expressed as mean ± SE (*n* = 5 per group). *P < 0.05, **P < 0.01, ***P < 0.001 versus Lean.

In addition to assessing tissue specific FA metabolism *in vivo*, we also examined the tissues capacity to oxidize FA by measuring palmitate oxidation rates in tissue homogenates. Independent of obesity status, the results demonstrate a range of 2 orders of magnitude in the capacity to oxidize FA across different tissues of the body, lowest in WAT and highest in the heart (Figure 4). In the obese animals there was only one tissue, BAT, where the fatty acid oxidation capacity was actually lower (−67%) than that observed in the lean animals, again consistent with the previously mentioned phenotypic difference. All other tissues of the obese animals examined showed either a similar capacity (heart, liver, cerebellum, and WAT) or a moderately enhanced (+35%) capacity (skeletal muscles) to oxidize FA compared with the tissues of the lean animals.

**Figure 5:**
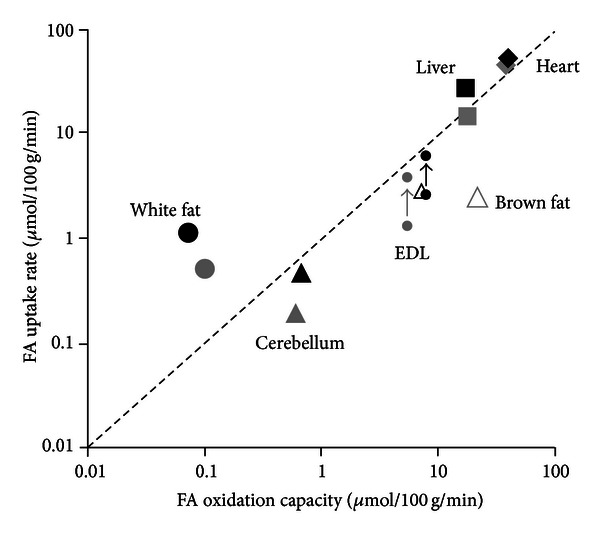
Relationship between FA uptake and FA oxidation capacity by individual tissues in lean (grey symbols) and obese (black symbols) Zucker rats. FA uptake has been calculated from *R*_f_* data (see Results section). The broken line indicates equality of uptake and oxidation capacity. FA uptake in the skeletal muscle, EDL (extensor digitorum longus), was assessed in the quiescent state and during sustainable twitch contractions: arrows indicate the effect of contraction.

To gain insight into the metabolic functionality of individual tissues, *in vivo* FA flux data are compared with the *ex vivo* estimates of FA oxidation capacity for individual tissues in Figure 5. Genuine rates of FA uptake (*y*-axis) were estimated from *R*_f_* values (Table 3) using procedures described in the Materials and Methods section. 

## 4. Discussion

In association with marked insulin resistance, nondiabetic obese Zucker rats exhibit substantial accumulation of triglycerides and lipid intermediates in non-adipose tissues, including liver and muscle [26–28]. This lipid accumulation could result from disturbances in tissue fatty acid uptake or metabolic fate including systemic oversupply, a locally enhanced ability to take up plasma FA or impairment of their oxidation. Which of these factors predominates in obese animals *in vivo* has not been resolved. In this study *in vivo* FA metabolism, both at the whole-body and individual tissue levels, was characterized in obese Zucker rats and compared to lean Zucker rats. Completely novel information concerning the flux of FA from plasma and its metabolic fate was obtained by applying a method based on the combined use of the non-*β*-oxidizable FA analogue tracer, ^3^H-R-BrP and ^14^C-palmitate [21]. 

The results demonstrate that oversupply of plasma FA is a major factor in the fatty acid overload of non-adipose tissues of the obese Zucker rat. We have shown in the current study and previously [29] that the rate at which FA enters the plasma (*R*_*a*_) in the fasting state in the obese animals is more than twice the rate of lean animals of the same age. The increased flux of fatty acid into the plasma has to be matched by a corresponding increase in flux of fatty acids into the tissues. Even after subtracting the fraction of *R*_*a*_ that disappears into the body fat, which is much greater in the obese animals, the remainder that supplies the non-adipose tissues of the body is still more than doubled in the obese compared to the lean animals, 14 versus 6 *μ*mol min^−1^, respectively, based on an average adipose tissue disposal equal to *R*_*fs*_ for epididymal fat (Table 4) and a body fat mass of 38% of body weight in obese versus 14% in lean animals (Table 1). 

The consequence of systemic FA oversupply is a general elevation of FA flux into non-oxidative metabolism in the tissues. Thus, in the obese animals the flux of plasma FA into storage metabolism was substantially increased in liver, skeletal muscle, WAT, and the heart compared to the corresponding fluxes in the lean animals (Tables 4 and 5). The group independent, linear relation between *R*_*fs*_ in quiescent muscles and plasma FA concentration (Figure 2) shows the importance of systemic FA in generating lipid overload in this metabolically important tissue, consistent with the old idea that FA metabolism is supply driven [30]. Our *in vivo* observations agree well with the results of previous *ex vivo* studies of skeletal muscle FA metabolism. Thus in both perfused hindlimbs [31] and incubated muscles [32] from normal rats, TG synthesis was found to be a linear function of the perfusate/media albumin-bound FA concentration, and synthesis rates were substantially higher in oxidative than glycolytic muscles. 

Tissue FA uptake is determined both by plasma and extracellular FA availability and the ability of the tissue to take up FA. An enhanced ability of skeletal muscle to take up FA has been documented to occur *in vivo* in skeletal muscle of high fat fed rats, which like the obese Zucker rat, exhibit insulin resistance associated with lipid accumulation [33]. There is also evidence suggesting that this could be the case in the obese Zucker rat. *Ex vivo* studies in perfused hind limbs from lean and obese Zucker rats where perfusate FA concentration had been equalized showed that in noncontracting muscle in the absence of insulin, FA uptake is augmented in obese compared to lean Zuckers [34, 35]. However this difference, which appears to be causally associated with the degree of translocation of putative FA transporter proteins [36], is condition dependent and can be abolished by insulin stimulation or contraction [37]. Moreover we found no evidence that the *in vivo* ability of muscle tissue to take up FA, assessed by the parameter *K*_f_*, is enhanced in the obese Zucker rats. While this is in contrast to the increased FA uptake reported in muscle of high fat fed rats [33], it should be noted that a comparable situation apparently exists in humans where fractional extraction of FA and perfusion of the leg were both determined to be similar in obese and lean volunteers in a classic study of Kelley and colleagues [38]. To summarize, our data suggests that at least under the conditions of this study, skeletal muscle FA oversupply in obese Zucker rats can be exclusively attributed to increased FA availability.

Another potential mechanism of muscle lipid overload is a reduction in local FA oxidation. However, our data provide no evidence that absolute rates of muscle FA oxidation *in vivo* were reduced in the obese compared with the lean Zucker rats. Information about the rate at which plasma FA enters the tissue and is immediately oxidized, rather than stored, was obtained by combining the tissue data derived from ^3^H-R-BrP, which reflects oxidative and non-oxidative fate, with that derived from ^14^C-palmitate incorporation into storage, reflecting non-oxidative metabolism only. Using this approach in the anesthetized preparation employed here, substantial levels of direct plasma FA oxidation were only in evidence in contracting muscles, including the beating heart and contracting diaphragm, as well as in contracting hindlimb muscles (Table 6). Most significantly, similar rates of direct oxidation of plasma FA were apparent in the working muscles of obese and lean animals. 

While the absolute rate of muscle FA oxidation is not reduced in the obese animals, this does not preclude an abnormality in the control of FA oxidation. One potential abnormality relates to the primary genetic defect in the obese Zucker, the loss-of-function mutation in the leptin receptor [39]. Leptin has been shown to acutely increase FA oxidation in skeletal muscle [20] and in the isolated heart [19]. On the basis of these effects, one would expect an inappropriately low rate of FA oxidation in these animals, relative to the prevailing plasma FA concentration. Indirect support for this mechanism in the liver has been provided in diabetic obese Zucker rats where transgenic overexpression of functional leptin receptor resulted in a remarkable reduction of the hepatic lipid content [39]. 

A confounding factor preventing direct comparison of oxidation rates in the obese compared with the lean animals is the higher prevailing plasma FA levels in the obese rats, which on its own would tend to drive higher rates of FA oxidation by simple mass action. To circumvent this issue we therefore studied the relationship between whole-body FA oxidation and FA availability by infusing the antilipolytic agent nicotinic acid in various doses to separate subgroups of animals (Figure 3). This revealed that indeed at comparable levels of plasma FA availability there was a lower rate of FA oxidation in the obese animals. Our *ex vivo* studies of palmitate oxidation seem to exclude the possibility that this results from a limitation in the capacity of tissues to oxidize FA. A much more likely explanation relates to the known elevation of tissue malonyl-CoA in the obese Zucker rats [40] which may be responsible by suppressing CPT I activity and diverting FA into storage as reviewed by [41]. In support of this, studies in our laboratory using pharmacological ACC inhibition in obese Zucker rats have shown normalization, relative to lean Zuckers, of both hepatic malonyl-CoA levels as well as ability to oxidize FA at the whole-body level (Oakes et al., unpublished observation). 

To investigate whether a defect in fatty acid oxidation was apparent at the subcellular level we assessed the capacity of a comprehensive range of tissues to oxidize palmitate. Skeletal muscle homogenates from the obese animals had a modest enhancement in the rate of palmitate oxidation (Figure 4) compared with the lean animal, in qualitative agreement with the recently reported increases in the activities of several important enzymes involved in mitochondrial oxidation in the muscle of obese compared with lean Zucker rats [36, 42]. All other tissues of the obese Zucker rat, with the exception of BAT, possessed a similar capacity to oxidize FA compared to the lean rats. Our data are consistent with the findings of Noland et al. [28] obtained using similar methods in liver, muscle, and heart of lean and obese Zucker rats. 

Overall the present data provide evidence that the excess lipid accumulation in non-adipose tissues, including skeletal muscle, of the obese Zucker rat is primarily due to an increased FA availability rather than a major intrinsic impairment in the ability to oxidize fatty acids. This deduction fits well with an *in vivo* study indicating equivalent mitochondrial oxidation capacity in skeletal muscle of diabetic Zucker rats compared to control rats [43]. Holloway et al. [36] also recently concluded, based on *ex vivo* studies, that intramyocellular lipid accumulation results from increased delivery of FA to the muscle cytosol rather than a defect in fatty acid oxidation. However, our *in vivo* findings diverge from those of Holloway et al. [36] in the major cause of the increased delivery (supply of FA to the myocyte versus enhanced plasma membrane transport) and these discrepancies likely relate to methodological differences in assessing fatty acid metabolism in the *in vivo* setting versus isolated *ex vivo* assessments. 

Comparing the estimates of *in vivo* FA uptake versus *ex vivo* fatty acid oxidation capacity provides information about the metabolic functionality of the individual tissues (Figure 5). Thus, the unique storage function of WAT is made apparent as it is the only tissue where FA uptake substantially exceeds oxidization capacity. By contrast, FA uptake in BAT lies well below its high capacity to oxidize, probably reflecting a rather low level of sympathetic activation under the physiological conditions of this study. Skeletal muscle also takes up much less FA than it is capable of oxidizing in the quiescent state, but sustainable twitch contractions causes uptake to approach oxidation capacity. In the beating heart, uptake and oxidation capacity were approximately matched, indicating that cardiac fatty acid oxidation is virtually maximal under the physiological conditions of this study. It is indeed remarkable that the intact heart seems to be able to extract FA from the plasma and oxidize it at approximately the maximal rate attainable *in vitro* where membrane barriers are absent and concentration gradients are presumably negligible. The points for the liver are also located on the line of equality of uptake and oxidation. However, unlike the heart, the liver exports a substantial fraction of the fatty acid taken up as plasma FA in the form of VLDL triglyceride and it also has a much greater capacity to transiently store FA as endogenous TG.

Based on ethical and practical considerations, whenever possible we use anesthetized preparations rather than conscious chronically catheterized animals for acute experiments. However one limitation is the fact that the skeletal musculature, of major importance for whole-body metabolism [44], in this situation is essentially inactive. To circumvent this problem we performed unilateral nerve stimulation to induce repetitive twitch contractions in a defined set of muscles in the lower hindlimb. This allows a comparison of substrate metabolism in specific contracting oxidative and glycolytic muscles of the stimulated leg with the corresponding contralateral resting muscles in the same animal. Our data show that contraction increases muscle FA uptake and oxidation without lowering the flux of FA into non-oxidative disposal. This implies that the contraction-induced increase in FA uptake is not simply the result of an elevated “pull” of fatty acids into mitochondrial *β*-oxidation. The latter is an important mechanism in the heart where increased respiration results in a fall in mitochondrial acetyl-CoA, in turn leading to a fall in cytosolic malonyl-CoA, relieving inhibition of carnitine palmitoyl transferase 1 (CPT I) and thereby increasing transport of fatty acyl-CoA into the mitochondria for oxidation [45]. A contraction-associated increase in skeletal muscle FA uptake without a reduction in FA storage is consistent with the involvement of a “push” mechanism and may be the result of enhanced membrane transport due to translocation of the fatty acid translocase (FAT/CD36) [46, 47]. 

While the methodology employed in this study offers a unique opportunity to study *in vivo* metabolism of plasma FA in a diverse range of tissues, an important limitation should be acknowledged: the method does not provide a complete assessment of overall tissue specific FA metabolism. Information is generated about the direct contribution of circulating FA to tissue specific FA metabolism but there are two significant alternative sources of FA: circulating TG and endogenous TG. The contribution of circulating TG to tissue specific FA metabolism is potentially large. Thus, using results from a previous study [29], rates of VLDL esterified FA secretion in the fasting state corresponded to 44% and 71% of the FA *R*_*a*_ for the lean and obese Zucker rats respectively. Since TG secretion must be matched by tissue TG uptake, the substantially elevated (~4-fold higher) VLDL TG secretion in the obese Zucker compared to lean age-matched Zuckers [29] is almost certainly also a major contributor to tissue lipid overload. However, little is known about the true *in vivo* tissue fate of this TG-FA and methods that can provide quantitative information about the contribution of both plasma TG-FA and FA are needed. A perhaps even more challenging issue is experimentally determining the contribution of the intracellular TG pool. While pulse labeling wash-out studies might be achievable for highly oxidatively active tissues like the heart [48], assessing TG turnover in tissues with modest rates of turnover is likely to be difficult.

## 5. Conclusions

In summary, our assessments of *in vivo* FA fluxes demonstrate that the major factor responsible for non-adipose tissue lipid overload in the insulin resistant obese Zucker rat is systemic FA oversupply. There was no evidence for a widespread impairment in the capacity of tissues from the obese animals to oxidize FA. At the whole-body level the absolute rate of FA oxidation was not reduced in obese Zucker rats under physiological conditions, but the marked increase in FA availability was not being matched by an equivalent elevation in FA oxidation, indicating some disturbances in fatty acid utilization. When conditions of equivalent FA availability were achieved *in vivo*, the obese animals exhibited a mild defect in FA oxidation compared to Lean Zucker rats. We showed that in skeletal muscle, fatty acid uptake associated with contraction was channeled into oxidation. By contrast, non-oxidative disposal of FA in skeletal muscle was heavily influenced by availability and remarkably did not appear to be diminished by a contraction mediated increase in local oxidative metabolism.

## Figures and Tables

**Table 1:** Body weights and plasma factors in lean and obese Zucker rats, Series 1.

	Lean	Obese
Body weight (g)	337 ± 21	512 ± 35***
Lean body mass (g)	291 ± 16	314 ± 18
Fat body mass (g)	47 ± 5	197 ± 18***
Plasma glucose (mM)	8.3 ± 0.4	12.4 ± 1.7*
Plasma insulin (nM)	0.4 ± 0.1	3.7 ± 0.9***
Plasma TG (mM)	1.2 ± 0.3	4.0 ± 0.6***

Data are mean ± SE (*n* = 6 per group).

*P < 0.05, ***P < 0.001 versus Lean.

**Table 2:** ^
3^H-R-BrP clearance (*K*_f_*) and ^14^C-P clearance into storage products (*K*_*fs*_) in individual tissues of lean and obese Zucker rats *in vivo*.

	*K*_f_* (mL 100 g^−1^ min^−1^)		*K*_*fs*_ (mL 100 g^−1^ min^−1^)	
	Lean	Obese	Lean	Obese
Cerebellum	0.46 ± 0.05	0.48 ± 0.04	0.87 ± 0.07	0.79 ± 0.05
Liver	37.5 ± 2.8	32.3 ± 2.3	45.9 ± 4.4	38.9 ± 2.8
WAT	0.69 ± 0.05	0.70 ± 0.06	1.6 ± 0.1	1.4 ± 0.2
BAT	7.2 ± 0.6	3.6 ± 0.5***	9.4 ± 1.0	7.8 ± 1.4
Heart	29.3 ± 2.8	16.5 ± 1.4**	15.5 ± 1.2	17.0 ± 3.3

*K*_f_* indexes the ability of the tissue to take up and utilize FA (for both oxidation and storage); *K*_*fs*_ indexes the ability to store FA. Data are mean ± SE (*n* = 6 per group).

*P < 0.05, **P < 0.01, ***P < 0.001 versus Lean.

**Table 3:** *K*_f_* and *K*_*fs*_ in individual hind leg muscles *in vivo* in lean and obese Zucker rats.

	*K*_f_* (mL 100 g^−1^ min^−1^)				*K*_*fs*_ (mL 100 g^−1^ min^−1^)			
	Lean		Obese		Lean		Obese	
	Con-Leg	Stim-Leg	Con-Leg	Stim-Leg	Con-Leg	Stim-Leg	Con-Leg	Stim-Leg
WG	0.76 ± 0.06	2.03 ± 0.23^†^	0.69 ± 0.04	1.86 ± 0.10^†^	1.87 ± 0.16	2.54 ± 0.12^†^	1.96 ± 0.18	2.35 ± 0.14
EDL	1.08 ± 0.08	3.38 ± 0.21^†^	1.06 ± 0.08	2.54 ± 0.21^†∗^	3.32 ± 0.31	4.26 ± 0.21^†^	4.26 ± 0.44	4.65 ± 0.23
RG	1.17 ± 0.07	2.58 ± 0.47^†^	1.39 ± 0.11	2.10 ± 0.17^†^	3.75 ± 0.23	5.08 ± 0.46^†^	4.48 ± 0.44	6.37 ± 0.54^†^
WQ	0.61 ± 0.05	0.66 ± 0.07	0.51 ± 0.02	0.52 ± 0.03	1.39 ± 0.10	1.42 ± 0.12	1.17 ± 0.06	1.16 ± 0.06
RQ	1.63 ± 0.13	1.54 ± 0.13	1.86 ± 0.13	1.76 ± 0.12	4.28 ± 0.38	4.06 ± 0.30	4.56 ± 0.21	4.14 ± 0.23

Sustained twitch contractions were induced in the lower leg muscles (WG white gastrocnemius, EDL extensor digitorum longus, and RG red gastrocnemius) but not in the upper leg muscles (WQ white quadriceps and RQ red quadriceps) by repetitive electrical stimulation of the sciatic nerve of one leg (Stim-Leg). The contralateral, control leg (Con-Leg) was not subjected to sciatic nerve stimulation. Results represent mean ± SEM (*n* = 6 per group). ^†^ *P* < 0.05 versus Con-Leg (paired *t*-test, effect of sciatic nerve stimulation), *P < 0.05 versus Lean (unpaired *t*-test, effect of obesity status).

**Table 4:** Indices of FA utilization (*R*_f_*) and FA incorporation into storage (*R*_*fs*_) in individual tissues of lean and obese Zucker rats *in vivo*.

	*R*_f_* (*μ*mol 100 g^−1^ min^−1^)		*R*_*fs*_ (*μ*mol 100 g^−1^ min^−1^)	
	Lean	Obese	Lean	Obese
Cerebellum	0.14 ± 0.01	0.32 ± 0.04***	0.26 ± 0.03	0.54 ± 0.07***
Liver	11.5 ± 1.4	21.8 ± 3.1**	13.7 ± 1.1	26.3 ± 3.5**
WAT	0.21 ± 0.02	0.47 ± 0.07**	0.47 ± 0.03	0.93 ± 0.16*
BAT	2.02 ± 0.14	2.37 ± 0.44	2.62 ± 0.26	5.28 ± 1.25
Heart	9.1 ± 1.3	10.2 ± 1.0	4.7 ± 0.4	10.1 ± 1.2***

*R*_f_* is an index of FA utilization rate (for both oxidation and storage) in the tissue. *R*_*fs*_ indexes flux of plasma FA into storage. Data are mean ± SE (*n* = 6 per group).

*P < 0.05, **P < 0.01, ***P < 0.001 versus Lean.

**Table 5:** *R*_f_* and *R*_*fs*_ in individual hind leg muscles in lean and obese Zucker rats.

	*R*_f_* (*μ*mol 100 g^−1^ min^−1^)				*R*_*fs*_ (*μ*mol 100 g^−1^ min^−1^)			
	Lean		Obese		Lean		Obese	
	Con-Leg	Stim-Leg	Con-Leg	Stim-Leg	Con-Leg	Stim-Leg	Con-Leg	Stim-Leg
WG	0.23 ± 0.03	0.63 ± 0.11^†^	0.47 ± 0.06*	1.22 ± 0.11^†∗^	0.57 ± 0.06	0.77 ± 0.23^†^	1.32 ± 0.20*	1.59 ± 0.23*
EDL	0.33 ± 0.04	1.04 ± 0.12^†^	0.72 ± 0.10*	1.67 ± 0.16^†∗^	1.01 ± 0.11	1.29 ± 0.10^†^	2.84 ± 0.42*	3.08 ± 0.28*
RG	0.36 ± 0.04	0.81 ± 0.17^†^	0.94 ± 0.13*	1.39 ± 0.15^†∗^	1.14 ± 0.10	1.56 ± 0.20^†^	3.01 ± 0.43*	4.24 ± 0.53^†∗^
WQ	0.19 ± 0.02	0.20 ± 0.02	0.34 ± 0.04*	0.35 ± 0.04*	0.42 ± 0.05	0.43 ± 0.04	0.77 ± 0.07*	0.77 ± 0.07*
RQ	0.50 ± 0.06	0.47 ± 0.06	1.24 ± 0.14*	1.19 ± 0.15*	1.29 ± 0.14	1.24 ± 0.13	3.04 ± 0.30*	2.79 ± 0.34*

Results represent mean ± SEM (*n* = 6 per group). ^†^ *P* < 0.05 versus Con-Leg (paired *t*-test, effect of sciatic nerve stimulation, within group), *P < 0.05 versus Lean (unpaired *t*-test, effect of obesity status, between group).

**Table 6:** *R*_*f*_ox__ (*μ*mol/100 g/min) in individual hind leg muscles in lean and obese Zucker rats.

	Lean		Obese	
	Con-Leg	Stim-Leg	Con-Leg	Stim-Leg
WG	0.30 ± 0.06	1.58 ± 0.34^†^	0.39 ± 0.23	2.88 ± 0.20^†∗^
EDL	0.22 ± 0.07	2.57 ± 0.31^†^	−0.23 ± 0.36	3.02 ± 0.44^†^
RG	0.20 ± 0.06	1.43 ± 0.44^†^	0.43 ± 0.37	0.83 ± 0.40
WQ	0.27 ± 0.03	0.32 ± 0.04	0.47 ± 0.11	0.50 ± 0.12
RQ	0.55 ± 0.08	0.52 ± 0.11	1.51 ± 0.31*	1.55 ± 0.30*

Results represent mean ± SEM (*n* = 6 per group). ^†^ *P* < 0.05 versus Con-Leg (paired *t*-test, effect of sciatic nerve stimulation, within group), *P < 0.05 versus Lean (unpaired *t*-test, effect of obesity status, between group).
